# Does the Cerebral Cortex Exploit High-Dimensional, Non-linear Dynamics for Information Processing?

**DOI:** 10.3389/fncom.2016.00099

**Published:** 2016-09-22

**Authors:** Wolf Singer, Andreea Lazar

**Affiliations:** ^1^Ernst Strüngmann Institute for Neuroscience in Cooperation with Max Planck SocietyFrankfurt am Main, Germany; ^2^Max Planck Institute for Brain ResearchFrankfurt am Main, Germany; ^3^Frankfurt Institute for Advanced StudiesFrankfurt am Main, Germany

**Keywords:** visual cortex, non-linear dynamics, synchrony and oscillations, plasticity and learning, recurrent networks

## Abstract

The discovery of stimulus induced synchronization in the visual cortex suggested the possibility that the relations among low-level stimulus features are encoded by the temporal relationship between neuronal discharges. In this framework, temporal coherence is considered a signature of perceptual grouping. This insight triggered a large number of experimental studies which sought to investigate the relationship between temporal coordination and cognitive functions. While some core predictions derived from the initial hypothesis were confirmed, these studies, also revealed a rich dynamical landscape beyond simple coherence whose role in signal processing is still poorly understood. In this paper, a framework is presented which establishes links between the various manifestations of cortical dynamics by assigning specific coding functions to low-dimensional dynamic features such as synchronized oscillations and phase shifts on the one hand and high-dimensional non-linear, non-stationary dynamics on the other. The data serving as basis for this synthetic approach have been obtained with chronic multisite recordings from the visual cortex of anesthetized cats and from monkeys trained to solve cognitive tasks. It is proposed that the low-dimensional dynamics characterized by synchronized oscillations and large-scale correlations are substates that represent the results of computations performed in the high-dimensional state-space provided by recurrently coupled networks.

## The evaluation and encoding of perceptual relations

Living systems have to establish models of the world in which they evolve in order to assure survival and reproduction because these models allow them to recognize and anticipate beneficial or detrimental constellations of inputs and to program adapted responses.

**Figure 1:**
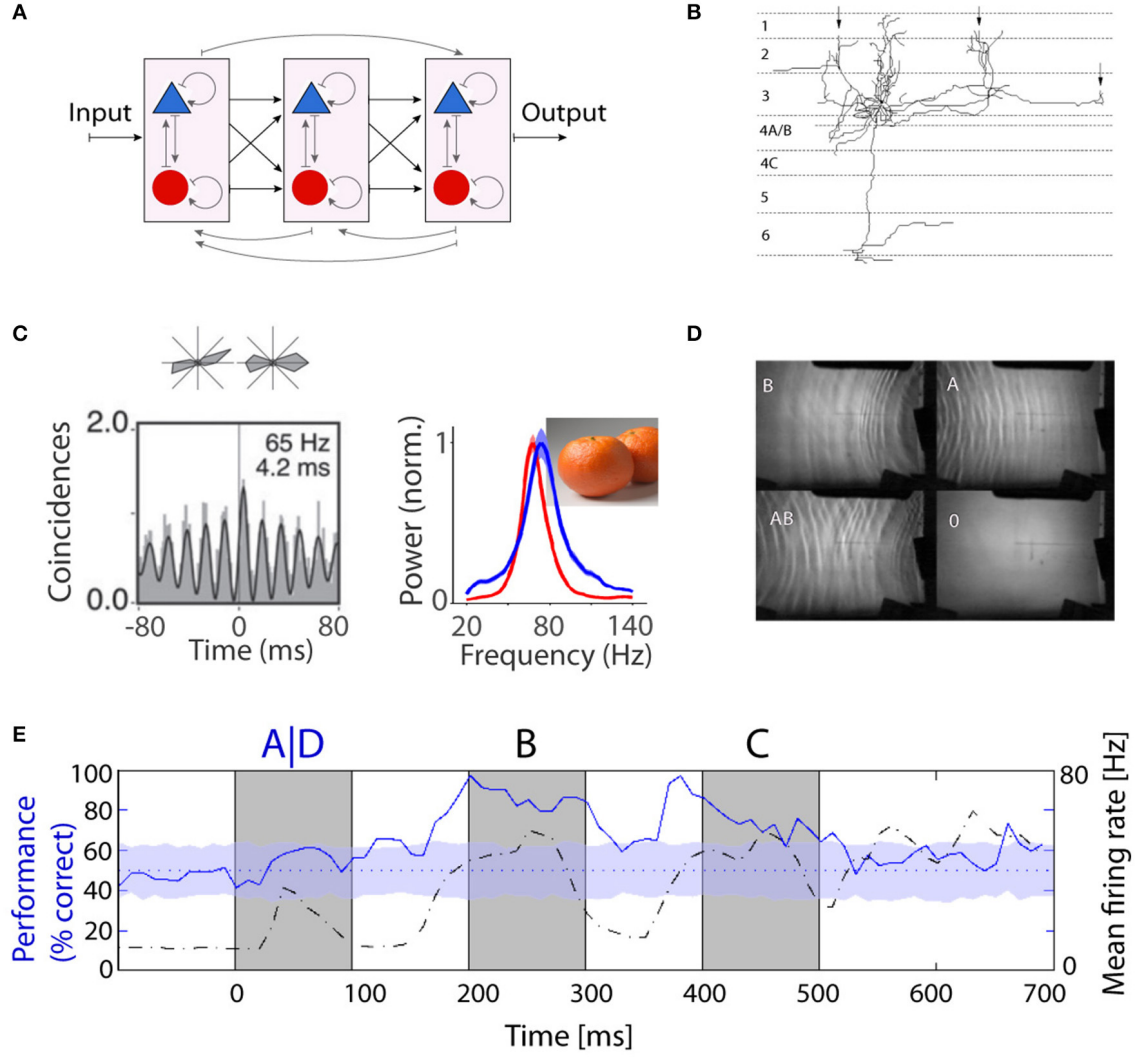
**Recurrent computation as a model for information processing in the brain. (A)** Schematic representation of a hierarchical structure consisting of a network of recurrent networks of excitatory and inhibitory units. So far, the majority of computational and experimental studies have focused on the feed-forward part shown in black. **(B)** Long-range lateral connections of a typical neuron in layer 3. The branches form local clusters in regions with similar functional properties (marked by arrows). Figure is adapted from McGuire et al. (1991). **(C)** Oscillations in monkey V1: Cross-correlogram for a pair of cells recorded from electrodes ~3 mm apart based on responses to grating stimuli (Lima et al., 2010) and natural image and corresponding LFP spectra during free viewing in two monkeys, red and blue (Brunet et al., 2015). **(D)** Practical demonstration of liquid computing: water tank with overhead projector, a perceptron was able to correctly map the XOR function based on interference patterns between waves. More details in Fernando and Sojakka (2003). **(E)** Fading memory in the primary visual cortex of anesthetized cat. Classifiers were trained to identify the first image in a sequence of three images (i.e., ABC vs. DBC). Classification performance (solid blue line) and population firing rates (dashed black line) are shown as a function of time from the presentation of the stimulus. Information about the first image could persist for long intervals of time (from Nikolic et al., 2009).

Establishing a good model of the world requires the detection of relevant and consistent relations between features of the environment and the efficient storage of these relations (rules). The simplest solution, found in virtually all neuronal systems, are relation encoding feed-forward circuits. Neurons tuned to respond to particular features of the environment converge on common target cells and these respond selectively to particular conjunctions of features if inputs and their gain are appropriately adjusted (Barlow, 1972). A particular relation among features gets encoded in the discharge rate of a neuron responding selectively to this relation. Because this neuron encodes always the same relation one talks about a “labeled line code.” In order to evaluate and encode combinations of relations (relations of higher order) this process of input recombination and gain adjustment is iterated across successive layers. This basic principle for the evaluation, encoding and classification of relational constructs has been implemented in numerous versions of artificial neuronal networks (Rosenblatt, 1958; Hopfield, 1987; DiCarlo and Cox, 2007; LeCun et al., 2015). The highly successful recent developments in the field of “deep learning” (LeCun et al., 2015), capitalize on the iteration of this principle in large multilayer architectures. As far as feed-forward connections are concerned, these artificial multilayer systems resemble the organization of sensory systems in the brain (Figure 1A). Marked differences exist, however, with respect to other essential features. Feed-back or recurrent lateral connections are missing in feed-forward artificial systems but are prominent in brains (Figure 1B) and often more abundant than feed-forward connections (Markov et al., 2014; Bastos et al., 2015). Moreover, the training mechanism used in technical systems for the supervised adjustment of the synaptic gain of feed-forward connections, the so called “back-propagation algorithm” is biologically implausible and differs radically from learning mechanisms implemented in brains (Feldman, 2012; Singer, 2016). While feed-forward architectures are well-suited to evaluate relations between simultaneously present features such as spatial relations, they are less apt to handle relations among temporally segregated events because they lack memory functions. Moreover, they are costly in terms of hardware requirements because the number of required units scales unfavorably with the number of represented relations.

A complementary way to detect and encode relations between signals is to evaluate temporal contingencies: If A consistently precedes B, it is likely to be the cause of B, if A and B often coincide the two events most likely have a common cause and if A and B are uncorrelated they are most likely unrelated.

The learning rules implemented in nervous systems are adapted to evaluate such temporal relations and to translate them into lasting changes of coupling. Both the traditional Hebbian rules (Hebb, 1949) and the more recently discovered mechanisms (Stiefel et al., 2005; Holthoff et al., 2006; Carvalho and Buonomano, 2011; Grienberger et al., 2015) evaluate temporal relations among converging inputs as well as between pre- and post-synaptic activity (spike timing dependent plasticity, STDP; Markram et al., 1997; Bi and Poo, 1998). According to the more refined STDP rule causal relations are evaluated and translated in synaptic gain changes. Connections strengthen between sending and receiving neurons if the former succeed to elicit post-synaptic spikes and connections weaken if the sender is active after post-synaptic spikes have been evoked by other inputs. This has two important implications: First, it implies that the precise timing of spikes matters in determining the occurrence and polarity of synaptic gain changes. Second, the mechanism subserving synaptic modifications not only evaluates simple covariations between pre- and post-synaptic firing rates, but also evaluates causal relations. It increases the gain of excitatory connections whose activity can be causally related to the activation of the post-synaptic neuron and it weakens connections whose activity could not have contributed to the post-synaptic response. Thus, temporal relations reflecting perceptual relations among events are evaluated by time sensitive mechanisms and converted into lasting changes of the coupling strength of interacting neurons. In this way, statistical contingencies among features of the sensory environment are translated into the synaptic weight distribution of neuronal networks.

This time sensitivity of synaptic plasticity mechanisms has deep implications for signal processing. If the known plasticity mechanisms were to be used for the storage of relations in general, these would have to be encoded in the temporal domain. Thus, for responses that lack temporal structure or are offset in time, mechanisms are required to endow them with precise temporal structure. In essence this implies that spatial relations have to be converted into temporal relations. Otherwise rather different and still unknown mechanisms of synaptic plasticity would have to be postulated to evaluate relations among responses lacking temporal structure.

## Mechanisms for the generation of temporally structured activity

Results from an initially completely different line of research suggest the existence of mechanisms capable of imposing temporal structure on neuronal activity and of making perceptually related responses coherent in time. It had been discovered with multisite recordings from the visual cortex that cortical circuits have a high propensity to engage in oscillatory activity and that these intrinsically generated oscillations can become synchronized, leading to correlated firing of the synchronously oscillating neurons. Of particular importance is the fact that this temporal coordination is dynamically regulated, is context sensitive and reflects meaningful relations among encoded features (Gray and Singer, 1989; Gray et al., 1989). These findings triggered a large number of studies on neuronal dynamics that provided evidence for intrinsic mechanisms capable of endowing neuronal responses with a temporal structure and to coordinate the timing of the discharges of distributed neurons with millisecond precision (for review see Singer, 1999; Engel et al., 2001; Fries, 2009; Uhlhaas et al., 2009). When neurons engage in oscillatory firing patterns in the range of the gamma frequency band their discharges become synchronized with a precision in the millisecond range (Figure 1C). This synchronization was observed not only between neurons located within the same cortical area (Gray et al., 1989), but also between cells in different cortical areas (Roelfsema et al., 1997), among cells in corresponding areas of the two hemispheres (Engel et al., 1991) and even between cortical and subcortical structures (Castelo-Branco et al., 1998; Brecht et al., 1999). Initially, the synchronization was seen as a relation defining mechanism mainly in the context of low-level visual processes such as feature binding and figure ground segregation. The reason was that synchronization probability reflected well the common Gestalt criteria for perceptual grouping and also reflected the architecture of the recurrent connections in the visual cortex that couple preferentially neurons coding for features which tend to be bound perceptually (Löwel and Singer, 1992). However, it soon turned out that synchronization of oscillatory activity is not confined to the visual system but a ubiquitous phenomenon (for review see Buzsáki et al., 2013). What makes these dynamic phenomena particularly interesting is the fact that they result from highly dynamic self-organizing processes that enable rapid reorganization of the temporal coherence of the responses of widely distributed groups of neurons. For this reason synchronization of oscillatory activity is now considered by many to serve a large number of different functions that have in common the requirement for temporal coordination of distributed neuronal responses. Examples are the enhancement of the saliency of responses (Fries et al., 1997; Biederlack et al., 2006), the dynamic formation of functional networks (Fries, 2005; Siegel et al., 2015; Deco and Kringelbach, 2016), the selection of responses by attention mechanisms (Fries et al., 2001a), the matching of top-down signals with sensory input (Bastos et al., 2015), the parsing of speech signals (Ding et al., 2016), and the definition of relations in the context of learning and memory (Siapas et al., 2005; Fell et al., 2011; Yamamoto et al., 2014; for review see Singer, 2016).

## Mechanisms supporting precise temporal coordination

Experimental and theoretical studies have clarified why oscillatory patterning of neuronal activity serves the adjustment of precise spike timing. Networks of inhibitory interneurons engage in oscillatory activity and convey periodic inhibition to excitatory cells that feed-back on the pool of inhibitory neurons (the PING mechanism, Kopell et al., 2000; Börgers and Kopell, 2003). The effect is that the spikes generated by pyramidal cells get concentrated around the depolarizing phases of the oscillations (König et al., 1995; Volgushev et al., 1998; Fries et al., 2007). During the hyperpolarizing phase of the oscillation cycle, incoming EPSPs have only a small chance to drive action potentials in the post-synaptic neuron because of the shunting and hyperpolarizing effect of the barrage of synchronously arriving IPSPs. The occurrence of post-synaptic responses can even be decoupled in time from the incoming EPSPs because of the special properties of NMDA receptors. Because of their high affinity to glutamate these bind the transmitter for more than a 100 ms but the associated ion channel opens only beyond a certain depolarization level due to the voltage gated magnesium block. Thus, glutamate released during the hyperpolarized phase of an oscillation cycle can only trigger AMPA receptor mediated EPSPs that are unlikely to reach threshold. However, when the membrane potential depolarizes and the magnesium block is removed, the NMDA channels open and the large inward currents usually trigger an action potential. Through this mechanism, the response to an EPSP arriving during the hyperpolarizing phase of an oscillation cycle can be delayed until the next depolarizing peak. In case of beta and gamma oscillations this delay can amount to up to 20 ms (Volgushev et al., 1998). Thus, the oscillatory modulation of the membrane potential concentrates spikes around the depolarizing peak of the oscillation cycle and causes synchronization of discharges between cells oscillating in phase. In addition, oscillations convert the strength of excitatory inputs into phase shifts of post-synaptic discharges relative to the oscillation cycle. Strong excitatory inputs elicit spikes earlier during the depolarizing cycle than weak inputs. Through this mechanism, known as phase precession (Huxter et al., 2003), rate-coded amplitude values are converted into a temporal code that is expressed in the timing of spikes relative to the oscillation cycle. Experimental evidence indicates that the phase offset of spikes relative to the oscillation cycle carries information (O'Keefe and Recce, 1993; Vinck et al., 2010; Womelsdorf et al., 2012). As has been shown in numerous theoretical studies, such temporal codes are advantageous for fast processing because information coded in spike timing can be read out without requiring temporal integration and thus much more rapidly than information encoded in discharge rates (for review, see Van Rullen and Thorpe, 2001; Fries et al., 2007). Once precise spike timing is achieved, transmission delays can be exploited in addition, in order to convert spatially structured input into a temporal representation (e.g., Wyss et al., 2003).

The integrative properties of neurons and the architecture of cortical networks also appear to be well-suited to deal with precise temporal codes. They are optimized for the distinction between temporally coherent and dispersed activity. A prominent feature of cortical connectivity is sparseness and as proposed by Abeles and confirmed later in simulation studies (Mainen and Sejnowski, 1995; König et al., 1996; Diesmann et al., 1999), such networks favor transmission of synchronized activity over transmission of temporally dispersed activity. Likewise, both the frequency attenuation of transmitter release at excitatory synapses between pyramidal cells and the adaptation of post-synaptic receptors, favor transmission of singular synchronized events, and decrease the effect of high frequency discharges. Sensitivity to synchronized (coincident) inputs is further enhanced by cooperative mechanisms in the post-synaptic dendrites. The voltage dependent sodium and calcium channels in the dendrites and their ability to convert high-amplitude EPSPs into regenerative spikes enhances further the coincidence sensitivity of cortical neurons because these large EPSPs are typically induced only by coincident input (Stuart and Häusser, 2001; Ariav et al., 2003). Finally, the membrane time constant of cortical neurons and hence the window for effective temporal integration of EPSP sequences is remarkably short when the neurons are in the up state, i.e., in their normal processing mode: this is due to the reduced membrane resistance caused by the balanced bombardment with EPSPs and IPSPs in the up state.

Thus, several lines of evidence indicate that cortical networks impose temporal structure on neuronal activity, relay temporally structured signals with high precision, and exploit temporal signatures for the encoding of relations. Thus, it appears that brains use two complementary strategies to encode and store relational constructs: One associates signals by having fixed anatomical connections converge onto conjunction units (labeled line coding). The other associates signals by rendering them coherent in time (binding by synchrony or temporal coding). The two strategies are complementary because timing sensitive synaptic plasticity can convert temporal codes into labeled line codes.

## Complex dynamics

As more laboratories engaged in multisite recordings, that permitted the analysis of the correlation structure of cortical dynamics, it became clear that oscillations with constant frequency sustained over long time intervals and synchronization of these oscillations with stable phase relations, occur only under specific stimulation conditions. Especially the high frequency oscillations in the beta and gamma frequency range were found to exhibit a much more complex and variable dynamics than reported in the early days of their discovery. In the visual cortex, the frequency of stimulus-induced oscillations increases with the energy and the complexity of the stimuli and with their motion speed (Gray et al., 1990; Kayser et al., 2003; Lima et al., 2011; Ray and Maunsell, 2015). The amplitude of stimulus-induced oscillations decreases with the complexity of the inducing stimuli and increases with attention and expectancy (Fries et al., 2001a; Lima et al., 2011). Moreover, in awake behaving animals the oscillations are usually transient, occur as brief bursts (Pipa and Munk, 2011; Lundqvist et al., 2016) and are often coupled with the phase of other oscillations that have lower frequency (cross frequency coupling, Canolty et al., 2010). Accordingly the pairwise correlations between oscillating cell populations are also highly variable. They are transient and exhibit phase shifts that vary over time (for review see Fries et al., 2001b; Maris et al., 2016).

It has been argued that this high degree of variability of oscillations and synchrony is incompatible with a functional role of these dynamic phenomena (Ray and Maunsell, 2015). This critique concerns both the initial postulate that temporal coherence serves to encode relations and the formation of assemblies in distributed coding regimes (Singer, 1999) as well as the extension of this concept known as the Communication Through Coherence (CTC) hypothesis (Fries, 2005). However, others have argued that variability and non-stationarity of the dynamics are necessary properties for flexible processing in order to comply with the speed and versatility of cognitive operations (Roberts et al., 2013) and with the requirement to configure on the fly functional networks on the fixed backbone of the cortical connectome (Deco and Kringelbach, 2016).

## A unifying concept

We propose a novel framework for cortical processing that attributes specific functions to the various manifestations of cortical dynamics, providing a cohesive interpretation of both low-dimensional states characterized by sustained frequency-stable oscillations and high-dimensional states with complex and rapidly changing correlation structure. The core of this proposal is that the cortex exploits the high-dimensional state-space provided by the non-linear dynamics of recurrently coupled networks in order to perform flexible and efficient computation. In this framework, emphasis is placed on the characteristic parameters of self-organizing complex systems with non-linear dynamics. These parameters include changes in correlation structure, the entropy and dimensionality of distributed activity, network oscillations, synchronization phenomena, and phase shifts. The proposed computational strategy is likely to account for a number of hitherto poorly understood functions: The encoding of temporal sequences, the storage of vast amounts of information about the environment in the networks of sensory cortices, the ultrafast retrieval of information in processes requiring comparison between input signals and stored knowledge, and the fast and effective classification of complex spatio-temporal input patterns.

Early theories of perception (von Helmholtz, 1867) have suggested that the brain interprets sparse and impoverished input signals on the basis of an internal model of the world. The information provided by this model is based on prior knowledge from visual experience (a form of memory) and is used to reduce redundancy, to facilitate segregation of figures from background, to bind signals evoked by features constituting a perceptual object, and to enable classification and identification. The complexity of the visual world is daunting. The store containing such an elaborate model must have an immense capacity in order to accommodate the vast number of statistical contingencies required for the interpretation of ever changing sensory input patterns. When primates, including humans, scan their visual environment, they change the direction of their gaze on average four times a second. Thus, the massive amount of prior knowledge required for the interpretation of a particular input pattern needs to be arranged in a configuration that renders it accessible within fractions of a second. The proposal is that these conditions can only be met if encoding, storage, and processing of information take place in the high-dimensional state-space provided by a complex system with non-linear dynamics.

## The hypothesis

Neocortex, especially its supragranular compartment, is ideally suited to provide such a high-dimensional coding space. It is a recurrently coupled network, whose nodes are feature selective and have a high propensity to oscillate. This network, so the assumption, provides the high-dimensional state-space required for the storage of statistical priors, the fast integration with input signals and the representation of the results in a classifiable format. Statistical priors are supposed to be stored in the functional architecture of long-range horizontal connections which are known to dominate this supragranular compartment (Gilbert and Wiesel, 1989; Stettler et al., 2002). Evidence suggests that these connections have been carefully crafted to match the statistical properties of visual scenes during the development of the visual system (Löwel and Singer, 1992; Smith et al., 2015) and continue to be plastic in adulthood (Eysel et al., 1998; Gilbert et al., 2009).

As the brain's spontaneous activity is constrained only by the brain's synaptic structure, this activity should reflect the dynamics of the structured network harboring the entirety of latent internal priors and exhibit high-dimensionality (note that this dynamics is a vast but constrained manifold inside the universe of all theoretically possible dynamical states). Input signals are supposed to trigger a cascade of effects: They drive in a graded way the feature sensitive nodes *and* thereby constrain the network dynamics. If the evidence provided by the input patterns can be easily interpreted by the computational circuitry, the network dynamics will collapse to a specific substate, corresponding to a particular perceptual experience. Such a substate is expected to have a lower dimensionality than the resting activity, exhibit specific correlation structures and be metastable due to reverberation among nodes supporting the respective substate. Because these processes occur within a very high-dimensional state-space, substates induced by different input patterns are well-segregated and therefore easy to classify. They can then either serve as input to the next cortical processing stage where new high-level interpretations emerge or they can be classified by local readout units that directly feed into executive centers. According to this concept, every cortical area has its own intrinsic computational circuitry and models visual concepts and their statistical relationships at that particular processing stage.

## Analogies from computational studies

In a much simplified version, the non-linear dynamics characteristic of recurrent networks are exploited for computation in AI systems, the respective strategies being addressed as “echo state, reservoir, or liquid computing” (Buonomano and Maass, 2009; Lukoševičius and Jaeger, 2009; D'Huys et al., 2012). The following Gedanken experiment illustrates the principles of reservoir computing. Objects impact at different intervals and locations in a reservoir of water and generate propagating waves whose parameters reflect the size, impact speed, and location of the object. The wave patterns fade with a time constant determined by the viscosity of the liquid, interfere with one another and create a complex dynamic state. This state can be analyzed by measuring at several locations in the reservoir the amplitude, frequency, and phase of the respective oscillations and from these variables a trained classifier can subsequently reconstruct the exact sequence and nature of the impacting “stimuli.” Fernando and Sojakka (2003) put these ideas in practice using an actual bucket of water and showed that the interference between waves on the water surface allowed a simple perceptron to solve the XOR problem (Figure 1D).

In artificial recurrent networks, the “reservoir” consists of non-linear units with random recurrent coupling that can maintain a certain level of intrinsic dynamics. In these models: (i) low-dimensional stimulus events are projected into a high-dimensional state-space where non-linear separable stimuli become linearly separable; (ii) The high-dimensionality of the state-space can allow for the mapping of more complicated output functions (like the XOR) by simple classifiers; (iii) Information about sequentially presented stimuli persists for some time in the medium (fading memory). As such, information about multiple stimuli can be integrated over time, allowing for the mapping of temporal functions. Together, these properties make artificial recurrent networks the state of the art models for complex sequence processing.

Building powerful artificial recurrent circuits is, however, non-trivial: a proper setting of structural parameters is essential in determining the complexity of the ensuing network dynamics and computational abilities. Artificial recurrent networks seem to function particularly well-near the edge of chaos, where their dynamics is rich yet predictable (Bertschinger and Natschläger, 2004). However, recurrent models that exhibit coexisting chaotic and locally stable trajectories are perfectly capable of keeping track of time on the order of seconds (Laje and Buonomano, 2013). In these models, recurrent plasticity can locally suppress chaos and substantially enhance computational power. Interestingly, more realistic implementations that mimic the connectivity patterns present in real cortical networks have computational advantages over models with random connections (Häusler and Maass, 2007). Moreover, recent simulation studies have shown that the performance of an artificial reservoir on sequence processing tasks is substantially improved if the recurrent connections are made adaptive and can “learn” about the feature contingencies of the processed patterns (Lazar et al., 2009; Hartmann et al., 2015).

## Experimental evidence

Because the connectivity of neurons in supragranular layers of the cerebral cortex resembles that of recurrent networks, we examined whether cortical dynamics exhibited some features of a “reservoir.” To this end we presented to anesthetized cats sequences of visual stimuli (letters and numbers), recorded with matrix electrodes simultaneously from a random sample of up to 124 neurons in primary visual cortex. We trained a linear classifier on short segments of activity (20 ms) based on a training set of population responses and then used the same classifier to identify the nature of the presented stimuli in a test set. The findings were encouraging (Nikolic et al., 2009). We found that (i) the information about a particular stimulus persists in the activity of the network for up to a second after the end of the stimulus, (ii) the information about sequentially presented stimuli superimposes so that two subsequent stimuli can be correctly classified sometime after the end of the second stimulus (Figure 1E) and (iii) the information about stimulus identity is distributed across neurons and encoded both in the discharge rate of the neurons and in the precise timing of the spikes.

Real cortical networks differ considerably from artificial systems in terms of complexity. Thus, one expects cortical networks to exhibit a much richer dynamics that provides additional options for computations. In most implementations artificial models are oversimplified, the recurrent connections used for reservoir computing are random and not adaptive. The nodes are usually simple versions of integrate and fire neurons and inhibition is often implemented by a single sign reversing element to prevent run away dynamics. By contrast, in the cerebral cortex the recurrent connections are highly specific due to genetic specification and experience-dependent shaping and have significant and heterogeneous conduction delays. Their layout reflects certain statistical contingencies of natural environments (Löwel and Singer, 1992; Smith et al., 2015). In addition, the nodes (neurons/columns) have complex integrative properties, are feature selective (Hubel and Wiesel, 1968) and have a strong propensity to oscillate (Gray et al., 1989). Likewise, the inhibitory interactions are mediated by a heterogeneous group of very selectively acting interneurons, whose connections are also susceptible to activity dependent long-term modifications (Moore et al., 2010). Finally, the recurrent networks in the supragranular compartment receive massive reentry loops from other cortical areas that are likely to bias dynamic states as a function of the higher-level computations performed in these other areas. Thus, if the cortex were to exploit principles of reservoir computing these marked differences would suggest that a “cortical reservoir” is highly structured, contains an internal and updatable model of the most relevant statistical features present in its input and can in addition be shaped by top-down influences from higher-level “reservoirs.”

## Predictions

Based on available data on the development and layout of the cortical connectome a number of experimentally testable predictions can be formulated: (i) The recurrent connections between neurons store an elaborate internal model which enhances perception based on prior experience. In vision this model is adapted to the co-occurrence statistics of visual features in natural images. (ii) The highly complex dynamics that evolve on the backbone of these recurrent connections provide the high-dimensional space for the accommodation of an immense repertoire of potential states. (iii) In response to input signals the initially unconstrained, high-dimensional internal network dynamic collapses into metastable subregions of the state-space. These regions are stimulus specific and reproducible across trials. (iv) These selected substates are distinguished by enhanced coherence (synchrony, covariance) and reduced variability. (v) The transition toward a stabilized lower dimensional substate should be optimal for natural stimuli, while noisy, ambiguous, and “unnatural” stimuli should be processed with more difficulty (a collapse to a substate may be slower, weaker, or the substate itself may carry less stimulus specificity or be more variable across trials). (vi) Importantly, the response dynamics to any stimulus should reflect the signatures of recurrent computation: the responses should be dynamic in time even if the stimulus is static and presented briefly. Present network activation states should contain a memory of past events. When multiple stimuli are presented sequentially an integration process should take place that is consistent with the underlying internal model: naturally occurring temporal transitions should be processed optimally. (vii) The collapse of the state-space into metastable subregions with increased coherence should promote their long-term stabilization by Hebbian modifications of recurrent connections and this, in turn, should facilitate the collapse to “familiar” substates in future operations and their readout by downstream processes. (viii) As a consequence, robustly consolidated substates should manifest themselves also in resting state activity and be detectable as “replayed” vectors or manifolds. (ix) In case the animal can predict the presentation of a stimulus, top-down signals should constrain the dynamic space in anticipation in order to speed up formation of specific substates. This should be reflected by anticipatory changes in dimensionality and correlation structure of network activity. (x) The proposed principle of information processing should be common to all cortical areas. Hence general principles of computation should be similar in different cortical areas but the mapping rules for the input and the nature of the stored internal model should differ.

Preliminary evidence is already available for some of these predictions. Developmental studies support the idea that the statistics of feature conjunctions in the outer world gets translated into cortical connectivity (Singer and Tretter, 1976) according to a Hebbian mechanism (Löwel and Singer, 1992; prediction i). In addition, the covariance structure of resting activity reflects the anisotropic layout of these connections (Gilbert and Wiesel, 1989; Löwel and Singer, 1992; Bosking et al., 1997; Fries et al., 2001b; Kenet et al., 2003), is modified by learning (Lewis et al., 2009; Kundu et al., 2013) and reveals hallmarks of an internal model of the environment (Berkes et al., 2011; predictions i and viii).

Ample evidence is also available for the ability of cortical circuits to engage in oscillatory activity in a wide range of frequencies and for stimulus dependent changes of correlations mediated by intracortical connections, both being hallmarks of recurrently coupled networks (for reviews see Singer, 1999; Buzsáki et al., 2013; prediction ii).

Much less is known about how the ensuing oscillatory responses depend on the particular properties of natural stimuli, both in the spatial and temporal domain (predictions iii–vi): e.g., how do particular Gestalt principles of grouping translate into informative neuronal dynamics and how noise or ambiguity affect the efficiency of this encoding (prediction v).

There are also indications that both sensory stimulation and top-down mechanisms related to attention might induce changes in the dimensionality of states (prediction ix), because they can enhance synchronized oscillatory activity in distinct frequency bands (Gray et al., 1989; Fries et al., 2001a; Churchland et al., 2010; Lima et al., 2011), however no direct analysis of dimensionality changes were performed in these studies. In Lima et al. (2011), the monkeys were presented with a sequence of three identical drifting gratings and were rewarded for responding if one of the gratings changed its orientation. A cue indicated to them whether the change was likely to occur for the second or third grating. The observation was that the “attended” grating evoked gamma band oscillations of much higher amplitude than the “non-attended” grating (Lima et al., 2011). Increased amplitudes of field potential oscillations indicate that either more neurons get entrained in the respective rhythm or/and that synchronization is more precise. Thus, the expectancy of having to respond to a particular stimulus changed the correlation structure of the activity induced by this stimulus toward enhanced coherence. In other terms, anticipatory top-down signals constrained the dynamics of an early visual area—most likely leading to a reduction of dimensionality.

Evidence is also available that points toward the fact that cortical circuits exhibit a fading memory of recent inputs. In Nikolic et al. (2009) information about a briefly presented stimulus could persist for up to 1 s and could superimpose with subsequent stimuli (prediction vi). Finally, we have preliminary evidence that repeated exposure to a set of images changes the response properties of populations of neurons in the primary visual cortex, such that stimulus classification improves over time: we observe changes in the dynamics of the network through the state-space that favor the segregation of responses into stimulus specific substates (prediction vii).

## Concluding remarks

Despite considerable effort there is still no unifying theory of cortical processing. As a result, numerous experimentally identified phenomena lack a cohesive theoretical framework. This is particularly true for the dynamic phenomena reviewed here because they cannot easily be accommodated in the prevailing concepts that emphasize serial feed-forward processing and labeled line codes. However, the cortical connectome with its preponderance of reciprocal connections and the rich dynamics resulting from these reciprocal interactions suggest that additional processing strategies are implemented. Here, we have proposed a concept that assigns specific functions to recurrent coupling and to features of the emerging dynamics. This concept is fully compatible with the robust evidence for labeled line codes and extends this notion by the proposal that precise temporal relations among the discharges of coupled neurons also serve as code for the definition of relational constructs both in signal processing and learning. We proposed a computational strategy that capitalizes on the high-dimensional coding space offered by reciprocally coupled networks. In this conceptual framework, information is distributed and encoded both in the discharge rate of individual nodes (labeled lines) and to a substantial degree also in the precise temporal relations among the discharge sequences of distributed nodes. The core of the hypothesis is that the dynamic interactions within recurrently coupled oscillator networks (i) endow responses with the temporal structure required for the encoding of context-sensitive relations, (ii) exhibit complex, high-dimensional correlation structures that reflect the signatures of an internal model stored in the weight distributions of the coupling connections, and (iii) permit fast convergence toward stimulus specific substates that are easy to classify because they occupy well-segregated loci in the high-dimensional state-space. The analysis of the correlation structure of these high-dimensional response vectors is still at the very beginning. However, methods are now available for massive parallel recording from large numbers of network nodes in behaving animals. It is to be expected, therefore, that many of the predictions formulated above will be amenable to experimental testing in the near future.

## Author contributions

WS and AL developed the research hypothesis and wrote the manuscript.

### Conflict of interest statement

The authors declare that the research was conducted in the absence of any commercial or financial relationships that could be construed as a potential conflict of interest.
